# Influences of race and clinical variables on psychiatric genetic research participation: Results from a schizophrenia sample

**DOI:** 10.1371/journal.pone.0284356

**Published:** 2023-04-12

**Authors:** Rose Mary Xavier, Yuktha Shanavas, Brian M. Britt, Wales T. George

**Affiliations:** 1 School of Nursing, University of North Carolina Chapel Hill, Chapel Hill, North Carolina, United States of America; 2 College of Medicine, SUNY Upstate Medical University, Syracuse, New York, United States of America; 3 College of Arts and Sciences, University of North Carolina Chapel Hill, Chapel Hill, North Carolina, United States of America; 4 Mental and Behavioral Health Services, Veterans Affairs Medical Center, Durham, North Carolina, United States of America; Duke University Medical Center: Duke University Hospital, UNITED STATES

## Abstract

Advances in genetics has led to a better understanding of both genetic and environmental contributions to psychiatric mental health disorders. But psychiatric genetics research is predominantly Eurocentric, and individuals of non-European ancestry continue to be significantly underrepresented in research studies with potential to worsen existing mental health disparities. The objective of this study was to examine factors associated with genetic study participation in a schizophrenia sample. The study sample was extracted from the Clinical Antipsychotics Trial of Intervention Effectiveness (CATIE) schizophrenia study which enrolled 1493 patients with chronic schizophrenia between the ages of 18–65 years and incorporated an optional genetic sub-study. Using a logistic regression model (N = 1249), we examined sociodemographic and clinical variables that were independently associated with the outcome i.e., participation in the genetic sub-study. The genetic sub-study had a lower proportion of Black (30% in genetic vs 40% in CATIE overall) and other race (4% vs 6%) participants. Increased severity of psychopathology symptoms (odds ratio [OR] = 0.78, p = 0.004) decreased the odds whereas better reasoning scores (OR = 1.16, *p* = 0.036) increased the odds of genetic study participation. Compared to Black participants, White participants were significantly more likely to participate in the genetic sub-study (OR = 1.43, p = 0.009). Clinical factors in addition to race significantly impact genetic study participation of individuals with chronic schizophrenia. Our findings highlight the need for future research that examines the interactive effects of race and clinical factors such as symptom severity on psychiatrically ill individuals’ choice to participate in genetics studies and to identify targeted strategies to increase equitable representation in psychiatric genetics research.

## Introduction

Genetic studies have overwhelmingly focused on individuals of European ancestry [1–3]. Despite concerted efforts to increase representation, individuals of African, Latin American, Hispanic, Indigenous and East Asian ancestry continue to be severely underrepresented in genetic studies [1]. For genetic studies of psychiatric and mental health disorders, this underrepresentation is quite pronounced [4, 5]. For example, the largest genome wide association study of schizophrenia (N = 320,404) comprised of 74.3% European samples 17.5% Asian, 5.5% African American and 2.5% Latino samples [6] whereas for bipolar disorder (N = 41,917) 100% of the study sample were European [7]. Scientific insights gained from one population does not carry over to another as differences in allele frequencies and genetic background confound the association of genes with the disorder or phenotype under study [8]. Subsequently, findings from predominantly European samples have poor predictive value in individuals of non-European ancestry [4]. Underrepresentation of individuals of non-European ancestry in psychiatric genetic studies thus has the potential to worsen existing mental health disparities [4]. Understanding factors that determine research participation is important to facilitate improved participation in psychiatric genetic studies and is critically important for underrepresented patient participants.

Patients’ research priorities often don’t align well with those of researchers and funding agencies [9]. What patients’ perceive as important can influence their research participation levels. Studies that have specifically examined factors affecting psychiatric and mental health research participation have identified fear of invasive procedures [10], the type of research (e.g., genetic studies, neuroimaging etc.), a lack of comprehension regarding research topic and study procedures, fear of treatment side effects, study safety and social stigma surrounding mental health illnesses as major factors that influence participation [11, 12]. The specific psychiatric diagnosis of the participant is also associated with their research participation. For example, compared to patients with depression, patients with schizophrenia report a lower approval of psychiatric research and a decreased readiness to participate in research using questionnaires and those requiring blood draws [10]. For both diagnostic groups, there is a lower level of willingness to participate in studies that are medication trials or that use neuroimaging techniques [10]. Patients with schizophrenia generally do not positively regard research if the methods were perceived as dangerous and involuntary [13].

Studies that have examined factors that impact research participation in psychosis and schizophrenia have primarily focused on the motivations and incentives that increase research participation. These studies show that the main factors incentivizing a schizophrenia patient’s participation included altruism (i.e., helping others and science) [13, 14] and monetary rewards [10]; how participants were informed and referred to research studies also influence research participation among patients with psychosis. For example, clear communication and a positive patient-clinician relationship were both key factors that increased research participation [15]. When the patient-clinician relationship is generally positive, patients were more likely to be referred to psychosis research studies [15].

Sociodemographic characteristics can influence who are exposed to opportunities for research participation as well as who are likely to participate in research studies. For example, gender and minority status influence how and where an individual access psychiatric care which in turn can influence recruitment into research studies [16]. Black and other persons of color experience multiple obstacles to active engagement in research including time constraints, lack of health insurance, lack of media access, and transportation issues to and from research sites [11, 17]. Black participants’ also have a higher level of mistrust regarding participation in genetic research compared to White participants regardless of their socioeconomic status and education levels [11, 18–20]. Black participants report concerns about the safety behind procedures as well as confidentiality about mental illness in families [11, 16]. But there is a dearth of knowledge regarding the social, demographic, and clinical factors associated with genetic research participation in the severely mentally ill such as those with schizophrenia and related psychosis.

The primary purpose of this study was to examine differences in sociodemographic and clinical characteristics of schizophrenia patients who chose to participate in a genetic study compared to those who chose not to. Specifically, we examined sociodemographic factors (such as age, gender, race) and clinical factors including psychopathology symptoms, insight and neurocognitive function known to influence participation in psychiatric research in general. As a secondary aim, we also investigated differences in longitudinal trajectories of symptoms and clinical outcomes between the participants who donated the DNA sample for the genetic study compared to those who did not.

## Methods

We obtained our study sample from the Clinical Antipsychotics Trial of Intervention Effectiveness (CATIE) schizophrenia study conducted between 2001 and 2004. Funded by the National Institute of Mental Health (NIMH), CATIE was a multiphase randomized controlled trial (N = 1493) that compared the effectiveness of four atypical antipsychotic medications—quetiapine, ziprasidone, olanzapine, and risperidone—against a typical antipsychotic, perphenazine, in patients with chronic schizophrenia between the ages of 18–65 years [21]. CATIE excluded those with treatment resistant schizophrenia. CATIE also incorporated an optional genetic sub-study that aimed to identify genetic risk variants associated with schizophrenia; ~51% (N = 738) of the CATIE sample participated in the genetic study. We accessed deidentified CATIE data from the NIMH Data Archive (NDA, https://nda.nih.gov/) after obtaining relevant data access permissions from the NDA and exempt ethical approval from Duke University IRB.

### Measures

*Genetic study participation* was measured based on if the participant donated a sample for the genetic sub-study–a dichotomous variable coded as ‘1’ for ‘Yes’ and ‘0’ for ‘No’.

*Psychopathology symptoms* were rated using the positive and negative syndrome scale (PANSS) [22] which has a total of 30 items with each item scored between 1 (absent) to 7 (extreme); the higher the scores the more severe the symptoms with total scores of <58 indicating mild illness, 58–75 as moderately ill, 76–95 markedly ill and 96–116 as severely ill. PANSS has 3 subscales—7 items assessing positive symptoms, 7 negative symptoms and 16 items focused on general psychopathology symptoms [23]. PANSS is a reliable and extensively validated scale [24] with good construct validity, and predictive validity [22]. We have previously reported a Cronbach’s α of 0.87 in our study sample [25].

*Insight* was measured by the insight and treatment attitudes questionnaire (ITAQ), a rater administered 11-item scale with good construct validity and reliability (Cronbach’s α ~ 0.95) [25], measures the participant’s attitude towards treatment i.e., treatment insight and their awareness of illness i.e., illness insight [26, 27]. ITAQ total scores range from 0 (indicating no insight) to 22 (indicating full awareness of illness), with higher scores indicating better insight. In this study we used the ITAQ total scores for analysis.

*Symptom severity* was assessed from both the patient and the clinician perspectives using the *Clinical global impression of symptom severity* (CGIS) [28]—the patient version measures the participant’s impression of their symptom severity. The clinician version is rated by clinicians to measure the global symptoms severity. Both items are scored from 1 to 7, with 1 being “Normal, not ill” and 7 being “Very severely ill”.

Severity of *depressive symptoms* in in the CATIE study was measured by the *Calgary depression rating scale* (CDRS), a valid and reliable measure for depression in schizophrenia patients [29]. CDRS measures nine mood symptoms which are scored from 1–4: less overall score indicates fewer depression symptoms.

*Neurocognition* measure was a composite score derived from working memory, verbal memory, processing speed, reasoning, and vigilance domain scores. Details on validity and reliability including interrater reliability and how the neurocognitive composite scores were calculated are published elsewhere [30].

*Health summary* was measured by the SF-12, a 12-item participant reported survey of mental and physical health. We used physical and mental health summary variables in our study; these variable scores range from 0 to 100. Higher scores indicate better general physical and mental health with scores <50 used for physical summary to determine a physical condition and a score of <42 indicating depression.

Patient symptoms and functioning over the preceding 4 weeks are measured using the *Quality of Life Scale* (QLS) [31], a measure with high sensitivity to subtle changes and effects of treatment [32]. It consists of 21 items, each rated on a 7-point primarily relying on clinician judgment.

The *MacArthur Competence Assessment Tool-Clinical Research* (MacCAT-CR) [33] was used to measure the capacity of the potential research participant to make competent decisions about research participation. The MacCAT-CR has good construct validity and reliability with an intraclass correlation ranging between 0.84–0.98 [34]. We used MacCAT 4 subscales in our analysis. *(1) Understanding*–the sum of the 13-item subset of the MacCAT-CR which pertains to a participant’s ability to understand provided information about a research project and its protocols. Scores can range from 0–26 with < = 15 suggests poor understanding. (*2) Appreciation–*the sum of the 3 items, can range between 0–6 and pertains to a participant’s ability to evaluate the consequences of study participation on themselves. *(3) Choice* is determined from the participant’s response to the item that pertains to a patient’s ability to clearly communicate a decision about their willingness to participate or not to participate in a research study. Scores can range between 0–2. *(4) Reasoning* is the sum of the 4 items with scores that can range between 0–8 and pertains to the patient’s ability to reason about their participation in a study by comparing alternatives and their consequences on themselves.

*Medication adherence* in CATIE was measured by the clinicians’ judgement based on data collected from patient report at all assessment visits, monthly pill counts and any other available information [21]. This information was coded on a scale of 1 to 4, with 1 being adherent always/most of the time, 2 for usually, 3 for sometimes and 4 for never/almost never. This was reverse coded for analysis.

### Statistical analysis

All analysis were implemented in R statistical software. We performed basic quality control including examining for duplicate entries on the data obtained from the NDA. We tested for assumptions of normality by examining data distributions of all variables included in our analysis. Variables in our analysis were all near normally distributed. Since there were missing data, we tested the missing mechanism using the Little’s test implemented in the R package *naniar* [35]. The data did not meet the assumption of missing completely at random (χ^2^ = 450, df = 292, *p* <0.001) and subsequently we performed multiple imputation to estimate missing data using the R package *MissForest* [36]. We conducted analysis on both the datasets–first on the original data with missing values and a second on the imputed dataset.

Primary aim: To examine if socio-demographic (age, gender, race) and clinical variables (psychopathology symptoms, insight, depression, neurocognition, quality of life, capacity for decision making and antipsychotic medication) that were independently associated with genetic study participation, we estimated a logistic regression model implemented in the R package *rms* [37] with blood sample donation status for genetic sub-study participation as the outcome variable using baseline/study entry data.

Secondary aim: To investigate if there were longitudinal changes in clinical variables between the overall and genetic sub-study groups, we estimated linear mixed models implemented in the R package *lme4* [38], assuming participant level random effects, with relevant clinical variables (psychopathology symptoms, insight, depression, neurocognition, general health, quality of life, capacity for decision making, illness severity) as the outcome variable and blood sample donation as the dependent variable adjusting for the effects of age, gender, race, and medication adherence across the study period.

## Results

Our overall study sample were 74% male and included 60% White participants, 35% Black participants and 5% were of other races—0.5% American Indian/Alaska Native, 2.3% Asian, 0.3% Hawaiian or Pacific Islander, 1.8% of mixed race, 0.1% unknown or not reported. Since the sample size for groups other than White and Black participants were small, we collapsed them into an ‘Other’ group for all analysis. Descriptive summaries of other variables are provided in Table 1. 52.4% of the overall sample participated in the genetic sub-study. Differences between groups that participated in the genetic study compared to those who chose not to are provided in Table 2. The genetic sub-study had a lower proportion of Black (30% in genetic sub-study vs 40% in CATIE overall) and Other (4% vs 6%) participants.

**Table 1:** Descriptive summary of variables at baseline.

Variables	Mean (SD)	Range	Interquartile range
Age in years	40.5 (11.1)	18–67	17
PANSS	75.9 (17.7)	31–140	23
Insight	18.1 (5.1)	0–22	7
Depression	4.5 (4.4)	0–22	6
Neurocognition (standardized)	0 (1)	-3.3–2.9	1.3
General physical health	48.2 (10.1)	13–68	14.5
General mental health	40.9 (11.7)	8–67	19
Quality of Life	2.7 (3.1)	0.2–5.9	1.5
Competency			
Understanding	23.4 (3.1)	0–26	4
Appreciation	4.5 (1.3)	0–6	2
Reasoning	5.7 (2)	0–8	3
Choice	2 (0.1)	0–2	0

**Table 2:** Socio-demographic characteristics by genetic sub-study participation.

	Participants who gave a DNA sample		Patients who did not give a DNA sample		Analysis		
Variable	Mean	SD	Mean	SD	t-test	df	p-value
Age(years)	40.9	11.0	40.1	11.2	-1.43	1441	0.153
Patient’s education(years)	11.6	3.5	11.4	3.7	-0.75	1411	0.456
Duration since first prescribed antipsychotic medication (years)	16.3	11.3	14.3	10.6	0.51	1415	0.611
Duration since first diagnosed (years)	14.0	10.9	16.2	10.8	-0.18	1416	0.860
	**Frequencies (proportion)**				**χ2**	**df**	**p-value**
Sex					0.61	1	0.437
Male	.73		.75				
Female	.27		.25				
**Race**					**23.1**	**2**	**1.0e-05**
Black	.30		.40				
Other	.04		.06				
White	.66		.53				
Employment					1.28	3	0.526
Full-time	.06		.07				
Part-time	.09		.08				
Did not work	.85		.85				
Marital Status					4.0	4	0.406
Divorced	.22		.19				
Married	.10		.12				
Never Married	.59		.60				
Separated	.06		.06				
Widowed	.02		.03				
**Medications switched at the study beginning**					**15.1**	**2**	**5.2e-04**
Newly treated	.24		.33				
Same Medication	.15		.13				
Switched Medication	.60		.53				
**Antipsychotic medication**					**13.6**	**3**	**3.6e-04**
None	.26		.35				
Second Generation	.57		.51				
First Generation	.07		.07				
First + Second generation	.10		.08				

Bolded values are statistically significant at p <0.05

**Fig 1:**
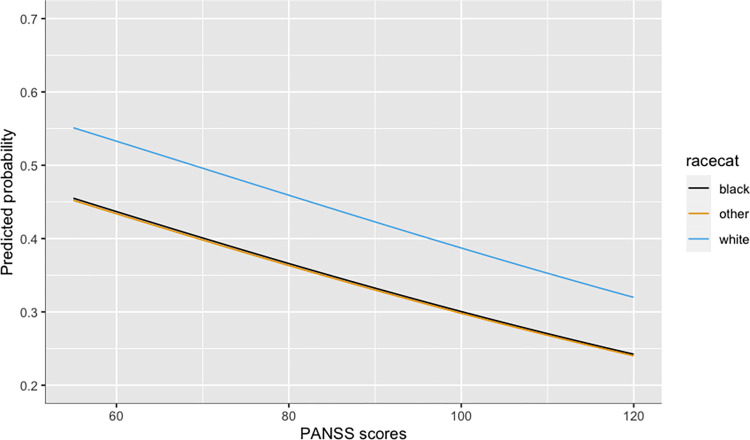
Predicted probabilities of genetic study participation from the logit model. Y-axis indicates probabilities and x-axis indicates psychopathology symptom scores measured by PANSS. At any level of psychopathology symptoms, Black and Other participants were less likely to participate in the genetic study compared to White participants.

For baseline data, the proportion of missing data for all variables of interest except neurocognition were less than 2%; neurocognition was missing for ~9% of the sample. We fitted a logistic regression model using maximum likelihood estimation to examine which sociodemographic and clinical variables at baseline were independently associated with genetic study participation. 95% confidence intervals and p-values were computed using the Wald approximation. Since the estimates for both the complete and imputed data were similar, we report and interpret our results from the complete data. Please see Table 3 for full results of the model. Our results show that increased severity of psychopathology symptoms assessed by PANSS scores significantly decreased the odds of whether a participant gave a DNA sample (odds ratio [OR] = 0.77, *p* = <0.001). A participant’s increased level of reasoning competency increased the odds of them giving a sample (OR = 1.16, *p* = 0.036). Compared to Black participants, White participants were more likely to give a blood sample for genetic study participation (OR = 1.43, *p* = 0.003). Please see Fig 1 for the predicted probabilities of genetic study participation for varying levels of psychopathology symptoms by race. Compared to participants who were not on any medications at baseline, those who on antipsychotic medications were more likely to provide a blood sample for genetic sub-study participation. But this association was only statistically significant for second generation antipsychotic medications (OR = 1.41, *p* = 0.013). Other variables included in the model were not statistically significant.

**Table 3:** Logistic regression results for baseline data (N = 1249).

	Variable	Complete data		
		Standardized Coefficient (log odds)	95% CIs	p-value
**Sociodemographic variables**	Age	0.06	-0.07, 0.19	0.350
	Gender (ref = female)	-0.04	-0.31, 0.24	0.787
	Education in years	0.01	-0.03, 0.04	0.743
	Race (ref = Black)			
	**White**	**0.39**	**0.13, 0.64**	**0.003**
	Other	-0.01	-0.57, 0.55	0.967
**Clinical variables**	Antipsychotic medication at baseline (ref = no med)			
	**2**^**nd**^ **generation**	**0.34**	**0.07, 0.61**	**0.013**
	1^st^ generation	0.16	-0.33, 0.65	0.522
	1^st^ + 2^nd^ generation	0.35	-0.08, 0.78	0.110
	Insight	0.05	-0.07, 0.17	0.418
	Depression	0.09	-0.03, 0.22	0.147
	**PANSS total**	**-0.26**	**-0.40, -0.12**	**<0.001**
	Neurocognition	-0.07	-0.21, 0.07	0.308
	Quality of life	-0.10	-0.25, 0.04	0.162
	MacArthur Competencies			
	Understanding	-0.12	-0.25, 0.009	0.068
	Appreciation	0.05	-0.09, 0.19	0.510
	**Reasoning**	**0.15**	**0.01, 0.29**	**0.036**
	Choice	0.07	-0.05, 0.19	0.246

Bolded values are statistically significant at p <0.05

Missing data proportions for longitudinal data are provided in the S1 Table. in CATIE, all structured assessments including the measures used in this study were done at baseline, 6, 12 and 18 months and at the end of the study. We included these timepoints for our longitudinal analyses as data was consistently collected on most participants. We fitted linear mixed models using restricted maximum likelihood estimation on both the original dataset and imputed dataset to examine longitudinal changes in 16 clinical variables by genetic study participation (Table 4). Estimations from original data were very similar to imputed data estimations, so we report and interpret results only from the original data. Compared to others, participants in the genetic sub-study longitudinally had significantly better insight (*β* = 0.63, *p* <0.05), were rated by clinician as less severely ill (*β* = -0.24, *p* <0.001) and had less severe psychopathology symptoms (*β* = -5.43, *p* <0.001) across the study period.

**Table 4:** Longitudinal changes in clinical variables by genetic sub-study participation.

Outcome Variable	N	Beta Coefficient for sample provided (ref = No)	p-value^a^
**Insight**	1309	**0.61**	**0.014** ^**b**^
Illness severity, patient rating	1316	-0.05	0.435
**Illness severity, clinician rating**	1316	**-0.20**	**8.19e-06**
Depression*	1319	0.20	0.306
**PANSS**	1320		
** Total**		**-4.63**	**1.57e-07**
** Positive**		**-1.40**	**4.89e-07**
** Negative**		**-0.98**	**0.001**
** General**		**-2.26**	**6.8e-07**
Neurocognition	1141	-0.05	0.340
General physical Health	1307	-0.37	0.410
General mental Health	1307	-0.09	0.858
Quality of life	1305	0.02	0.659
MacArthur Competency Subscales	1230		
Understanding		-0.21	0.148
Appreciation		-0.00	0.998
Reasoning		0.04	0.652
Choice		0.01	0.221

Note: Genetic study participation indexed as sample provided is the explanatory variable of interest for all outcome variables. Gender, age, race, medication adherence and medication adherence x time (interaction) were included as covariates in all models; these estimates are not provided in the table.

*Imputed model was significant for depression with beta 0.44, p = 0.016

^a^ Uncorrected p-values reported

^b^ not significant at the Bonferroni corrected p-value threshold of <0.003 for 16 linear mixed models reported in this table.

## Discussion

Our study examined sociodemographic and clinical characteristics of genetic study participation using a relatively large sample of individuals with chronic schizophrenia. We report that among individuals with chronic schizophrenia, clinical factors (severity of psychopathology symptoms, reasoning abilities and illness severity in general) and sociodemographic factors such as self-reported race significantly influence genetic study participation. Our finding that individuals with severe symptoms and poorer reasoning scores (which are strongly correlated with cognitive dysfunction in schizophrenia; please refer to S1 Fig) were less likely to participate presents a challenge for investigations into the genetic contributors to schizophrenia. Individuals with the most severe illnesses can be highly informative samples not just to understand illness etiology, but also to develop tailored interventions that improve clinical outcomes for patient subgroups with severe illnesses. But participation in research is imperative to ensure benefits from research. Our study findings highlight the need to actively engage and recruit severely ill individuals. It is also important to consider factors that support retention as psychiatric genetic discoveries progress towards clinical applications—discovery studies generally require a single contact whereas clinical genetic studies often need to be longitudinal. But there is a critical gap in our knowledge as to the why there are differences in initial participation as well as retention rates in individuals with schizophrenia. Future studies should be designed to facilitate this type of inquiry and must engage individuals with lived experience not just as research participants but as research partners and investigators. Approaches such as community based participatory research has demonstrated value in psychiatric services research and should be considered in psychiatric and mental health genetics research.

Psychiatric genetics research in general (not just schizophrenia genetics research) has a significant underrepresentation of individuals of non-European ancestry [4, 6, 39]. Despite concerted efforts (such as through policy initiatives by the NIH), this underrepresentation continues to widen [2, 39, 40]. Research findings from the general population show that there is a historically justified lack of trust among Black individuals when it comes to genetics research participation [11, 20]. The general mistrust in biomedical research stems not just from historical events e.g., the Tuskegee syphilis study, but is strengthened by discriminatory experiences Black Americans continue to face within the current health care system [41]. There are no studies that have examined genetics research participation specifically in severely psychiatrically ill groups of non-European ancestry. A study that examined racial differences in participation for a genetics of nicotine dependence study showed that willing to participate was not a significant barrier once the racial minority groups were reached by targeted recruitment and engagement [42]. It is unknown if such targeted recruitment and engagement would suffice to improve participation of individuals who have a severe mental illness or those with more severe symptoms in general. Studies of race and ethnic differences in utility of genetic findings and ethical concerns of individuals with severe mental illnesses are scant; most of them predominantly including White individuals [43–45]. Our study findings demonstrate the gap in schizophrenia genetic studies, but extant literature shows that this is also the case for other mental illnesses such as bipolar disorder [39]. Race is not a valid genetic variable, but it is a valid and important social variable that influences important mental health outcomes and as such deserves due consideration in the design stage itself. This is important not just to ensure that research benefits are equitably distributed, but is a necessity to accurately quantify genetic risk [5].

Our study findings show a statistically significant association of genetic study participant by the type of antipsychotic medication at baseline—those who were on second generation antipsychotic medications alone were more likely to participate. Second generation medications are the first line treatment for schizophrenia and patients who are on second generation medications tend to be more treatment responsive or are likely to have fewer years of treatment since initial diagnosis [46]. Our study design and data does not allow for further inquiry into this but suggest the need for further investigation. The CATIE sample though a nationally representative is a US based sample; further studies are warranted to examine if these findings are similar in other populations. Our study findings are also limited in that, the design allows us to only examine initial participation and not retention, which is critically important for clinical genetics studies. Nevertheless, our study highlights the need to understand the intersectional influences of clinical and sociodemographic factors in an individual’s decision to participate in a genetic study. The genetic study was optional in CATIE; this allowed us to examine factors influencing participation for schizophrenia patients, but such studies involving diagnosed individuals are critically warranted in other psychiatric disorders including major depressive, bipolar disorders and eating disorders among others.

## Supporting information

S1 Table N of complete data for relevant variables across time points. (DOCX)

S1 Fig Correlogram of relevant variables from baseline. Measures for variables are included in parenthesis. CDRS–Calgary Depression Rating Scale; CGIS–Clinical Global Impression of Symptom Severity; ITAQ–Illness and Treatment Attitude Questionnaire; MacCAT-CR–MacArthur Competence Assessment Tool- Clinical Research; PANSS–Positive and Negative Syndrome Scale; SF-12–12-item Short Form Survey; QLS–Heinrich-Carpenter Quality of Life Scale.
(DOCX)
