# Supplementary material for: Influences of race and clinical variables on psychiatric genetic research participation: Results from a schizophrenia sample
Source: PLoS One. 2023 Apr 12;18(4):e0284356. doi: 10.1371/journal.pone.0284356 (PMC10096269; doi:10.1371/journal.pone.0284356)
Supplement: S1 Table — (DOCX) [file pone.0284356.s001.docx]

**S1 Table. N of complete data for relevant variables across time points**

| **Variables** | | **Timepoints** | | | |
| --- | --- | --- | --- | --- | --- |
|  | | **Four** | **Three** | **Two** | **One** |
| **Insight** | | 765 | 941 | 1117 | 1328 |
| **Illness severity (patient)** | | 777 | 949 | 1117 | 1335 |
| **Illness severity (clinician)** | | 775 | 951 | 1127 | 1335 |
| **Depression** | | 785 | 952 | 1124 | 1338 |
| **PANSS** | | 786 | 953 | 1128 | 1339 |
| **Neurocognition*** | | - | 539 | 824 | 1165 |
| **General physical health** | | 757 | 934 | 1113 | 1325 |
| **General mental health** | | 757 | 934 | 1113 | 1325 |
| **Quality of life** | | 764 | 939 | 1112 | 1324 |
| **Competencies*** | **Understanding** | - | 628 | 964 | 1253 |
|  | **Appreciation** | - | 628 | 964 | 1253 |
|  | **Reasoning** | - | 628 | 964 | 1253 |
|  | **Choice** | - | 628 | 963 | 1253 |

*****These variables only have 3 timepoints for consistent measurements for most participants.
