# Supplementary material for: Influences of race and clinical variables on psychiatric genetic research participation: Results from a schizophrenia sample
Source: PLoS One. 2023 Apr 12;18(4):e0284356. doi: 10.1371/journal.pone.0284356 (PMC10096269; doi:10.1371/journal.pone.0284356)
Supplement: S1 Fig — Measures for variables are included in parenthesis. CDRS–Calgary Depression Rating Scale; CGIS–Clinical Global Impression of Symptom Severity; ITAQ–Illness and Treatment Attitude Questionnaire; MacCAT-CR–MacArthur Competence Assessment Tool- Clinical Research; PANSS–Positive and Negative Syndrome Scale; SF-12–12-item Short Form Survey; QLS–Heinrich-Carpenter Quality of Life Scale. (DOCX) [file pone.0284356.s002.docx]

**
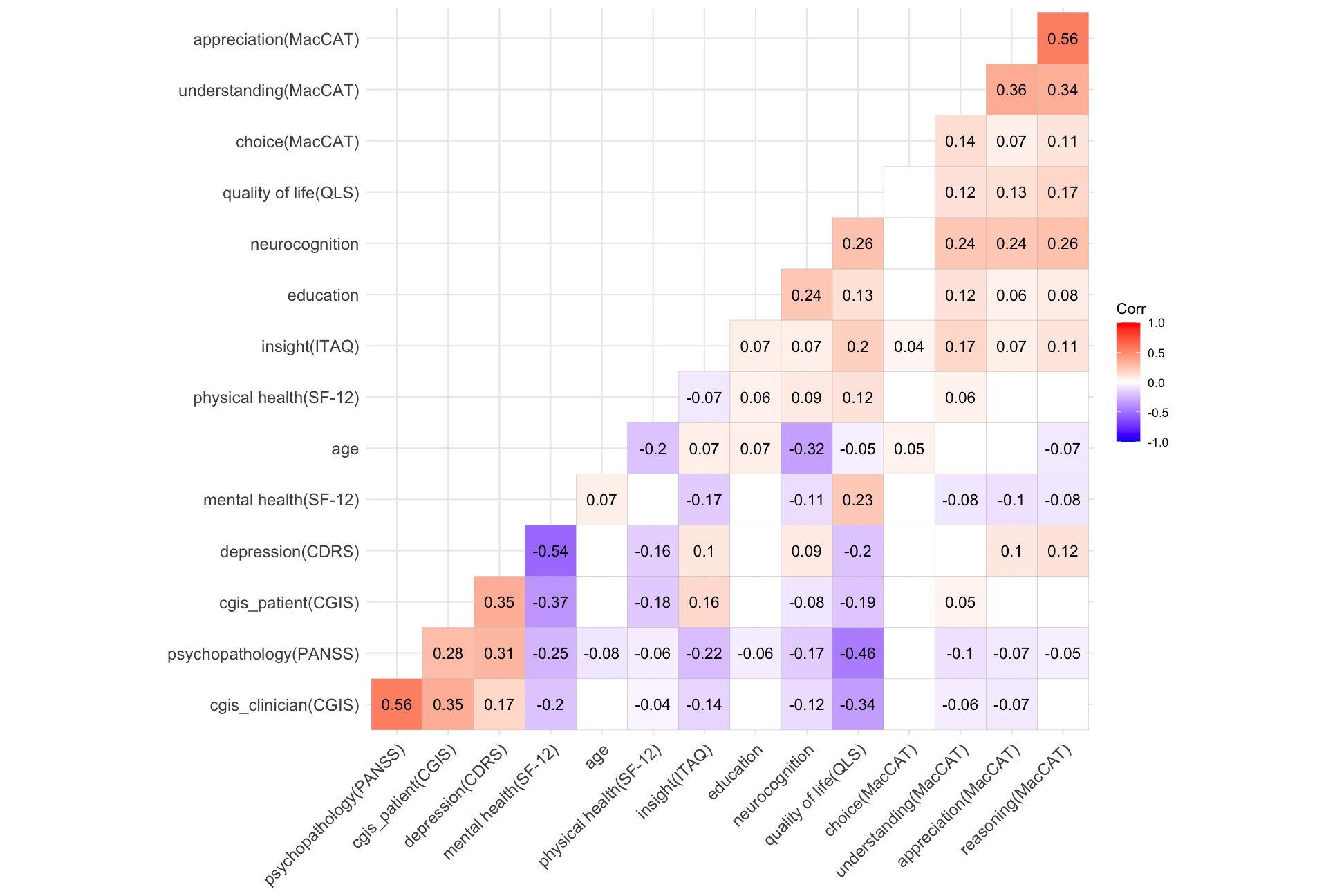
**

**S1 Fig. Correlogram of relevant variables from baseline**. Measures for variables are included in parenthesis. CDRS–Calgary Depression Rating Scale; CGIS–Clinical Global Impression of Symptom Severity; ITAQ–Illness and Treatment Attitude Questionnaire; MacCAT-CR–MacArthur Competence Assessment Tool- Clinical Research; PANSS–Positive and Negative Syndrome Scale; SF-12– 12-item Short Form Survey; QLS­–Heinrich-Carpenter Quality of Life Scale.
